# Impact of Sonication Fluid Cultures on Prosthetic Joint Infection Diagnosis and Management: A Microbiology-Driven Evaluation Using Infectious Diseases Society of America and European Bone and Joint Infection Society Criteria

**DOI:** 10.1093/cid/ciaf391

**Published:** 2025-07-18

**Authors:** Anas Zouitni, P Koen Bos, Marius Vogel, Peter Croughs, Lennert Slobbe, Paul-Emile Claus, Jakob van Oldenrijk, Ewout S Veltman, Erlangga Yusuf

**Affiliations:** Department of Orthopedic Surgery and Sports Medicine, Erasmus Medical Center, Rotterdam, The Netherlands; Department of Orthopedic Surgery and Sports Medicine, Erasmus Medical Center, Rotterdam, The Netherlands; Department of Medical Microbiology and Infectious Diseases, Erasmus Medical Center, Rotterdam, The Netherlands; Department of Medical Microbiology and Infectious Diseases, Erasmus Medical Center, Rotterdam, The Netherlands; Department of Medical Microbiology and Infectious Diseases, Erasmus Medical Center, Rotterdam, The Netherlands; Department of Clinical Laboratory, Imelda Hospital, Bonheiden, Belgium; Department of Orthopedic Surgery and Sports Medicine, Erasmus Medical Center, Rotterdam, The Netherlands; Department of Orthopedic Surgery and Sports Medicine, Erasmus Medical Center, Rotterdam, The Netherlands; Department of Medical Microbiology and Infectious Diseases, Erasmus Medical Center, Rotterdam, The Netherlands

**Keywords:** prosthetic joint infection, sonication, microbiology, diagnosis, antibiotic choice

## Abstract

**Background:**

Most studies investigating the role of sonication fluid culture on periprosthetic joint infections (PJIs) focused on diagnostic accuracy, without assessing its potential impact on patient management. This study aims to evaluate how the use of sonication fluid culture may alter the diagnosis and management of PJI.

**Methods:**

Included were 429 patients (mean age [SD], 68 [11] year; 65% male) who underwent explantation of a knee or hip prosthesis due to suspected PJI or aseptic causes. Because the Infectious Diseases Society of America (IDSA) PJI criteria do not include sonication in the diagnostic definition, we compared sonication fluid culture results with intraoperative tissue cultures patient-per-patient. In contrast, the European Bone and Joint Society (EBJIS) criteria include both sonication and tissue cultures. For EBJIS-confirmed criteria, we calculated the proportion of cases meeting the sonication fluid culture criterion of >50 colony-forming units (CFU)/ ml, and the number of cases where sonication identified additional microorganisms than tissues.

**Results:**

Among 95 PJI cases defined by IDSA criteria, sonication fluid culture would not alter patient management in 61 (64.2%) cases, as findings were fully concordant with tissue cultures. In 8 (8.4%) cases, sonication detected an additional virulent microorganism that could influence antibiotic selection. According to EBJIS criteria, 113 cases were classified as confirmed; 50 (44.2%) met the definition based solely on a positive sonication fluid culture >50 CFU/ml. In 10 of these 50 patients (20%), sonication influenced antibiotic selection by identifying additional low-virulence microorganisms.

**Conclusions:**

Sonication fluid culture may influence clinical management in approximately 8% to 20% of PJI cases.

## Teaser

Sonication fluid culture can potentially influencing clinical management (ie, diagnosis and antibiotic selection) in 8% to 20% of periprosthetic joint infection patients especially by identifying additional microorganisms.

## Graphical Abstract

**Graphical Abstract:**
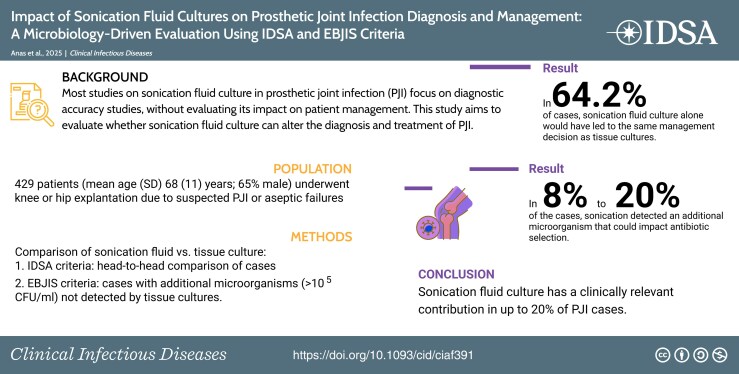
This graphical abstract is also available at Tidbit: https://tidbitapp.io/institutional-portal/clinical-infectious-diseases/tidbits/impact-of-sonication-fluid-cultures-on-prosthetic-joint-infection-diagnosis-and-management-a-microbiology-driven-evaluation-using-idsa-and-ebjis-criteria/update

Prosthetic joint infection (PJI) is a serious complication of joint replacement surgery [1]. To improve PJI diagnostic, sonication of removed implants have been introduced [2, 3]. Numerous studies have investigated the values of sonication fluid cultures. The studies are primarily diagnostic accuracy studies, focusing on calculating the sensitivity and specificity of sonication fluid cultures when compared with clinical reference standards. The findings from these studies have been inconsistent and often contradictory [4–8]. This uncertainty has been reflected in a European-wide survey reported that only half of participating laboratories routinely performed sonication in their laboratories [9].

The differences between sonication culture and tissue culture are expected, given that they are fundamentally different methods. Sonication aims at dislodging biofilm from a foreign body [10], and sonication fluid culture differs from tissue culture in terms of inoculum, culture media, and the need of thresholds to interpret positive results. Due to emphasis on sensitivity and specificity, diagnostic accuracy studies often overlook the differences in microbial findings between the 2 methods. Sonication culture may identify different microorganisms compared to tissue culture. In some cases, it can detect additional organisms that tissue culture fails to identify in polymicrobial infections. Conversely, sonication may also miss microorganisms that are detected in tissue culture. These discrepancies are clinically important, as they not only affect to define PJI cases but also influence the patient management and selection of appropriate antibiotics.

Analyzing the role of sonication fluid culture is further complicated because the 2 most common criteria for defining PJI used different approaches. The Infectious Diseases Society of America (IDSA) guideline published in 2012 [11], recommends submitting explanted prostheses for sonication culture, but it does not include the results in its definition of PJI. In contrast, the more recent European Bone and Joint Infection Society (EBJIS) criteria [12] includes not only sonication but also incorporates specific thresholds for interpreting sonication culture, that is, > 1 colony-forming unit (CFU)/mL of any organism as one of the criteria for likely infection, and >50 CFU/mL alone is a criterion sufficient to indicate confirmed infection. An additional nuance is that the IDSA guidelines clearly consider the isolation of a virulent microorganism sufficient for diagnosing PJI, whereas the EBJIS criteria only reference this distinction in a footnote.

Clearly, there is a need for a more in-depth study into the role of sonication fluid culture on the clinical definition and management of PJI. Therefore, the aim of this study is to evaluate the impact of sonication fluid culture on the diagnosis and management of PJI, using both the IDSA and EBJIS criteria, with specific consideration of CFU thresholds and polymicrobial findings.

## PATIENTS AND METHODS

### Setting and Study Population

The study population included all consecutive patients who had knee or hip prosthesis explanted due to septic or aseptic causes at the department of Orthopedic Surgery of Erasmus University Medical Center between February 2012 and May 2021. This center has 1300 beds and is 1 of 7 tertiary hospitals in the Netherlands. By protocol, 3–6 intraoperative tissue cultures were taken, and all explants were routinely sent for sonication procedure, regardless of whether infection or aseptic failure was suspected.

Patient data were anonymized, and because the study used recorded data, it was exempt from the medical ethical committee approval under Dutch law (“niet WMO plichtig”).

We collected demographic data and variables needed to diagnose PJI according to IDSA and EBJIS criteria. As far as possible, clinical, laboratory, and radiographic parameters were considered if obtained within 8 weeks prior to joint explantation. We classify symptoms lasting <4 weeks as acute and longer as chronic as described before [13]

### Microbiological Procedures

Explanted prostheses were collected in a sterile polypropylene container, sent to our clinical microbiology laboratory, and processed within 4 hours after receipt. A 0.9% sterile NaCl solution was added to the container until 90% of the explant surface is covered. The container was vortexed for 30 second and then placed in a sonication bath (Bactosonic, Bandelin GmbH, Germany) at frequency of 40 kHZ and power 200W for 1 minute. The resulting sonication fluid was centrifuged 3200 rpm for 15 minutes. Then, 100 μL of the resuspended pellet was inoculated onto blood and chocolate agar for aerobic culture, and BD™ brucella agar for anaerobic culture. Additionally, 10 mL of the resuspended sonication fluid was inoculated into aerobic and anaerobic blood culture bottles (Bactec, Becton and Dickinson, Sparks, USA). All agars and blood cultures were incubated for 14 days. The limit of quantification was 10 CFU/mL for growth on agar, and 1 to 10 CFU/mL when growth was detected only in blood culture bottles. Perioperative tissue samples were collected from various locations around the prosthesis using separate sterile forceps. Each sample was placed in separate sterile containers and sent to the laboratory. They were inoculated onto the same agars as above, with the addition of MacConkey agar. Synovial fluid was inoculated onto aerobic pediatric blood agar and the same agars as used for tissues, under identical incubation conditions and durations.

We considered the following as virulent microorganisms [14]: *Staphylococcus aureus*, *Staphylococcus lugdunensis*, *Enterococcus* spp., beta-hemolytic streptococci, *Streptoccocus dysgalactiae*, Enterobacterales, and *Pseudomonas aeruginosa*. *Cutibacterium acnes*, coagulase negative staphylococci, *Corynebacterium* spp., anaerobes, and other microorganisms not commonly associated with PJI for the purpose of this study were considered as low-virulence microorganisms. *Microccocus* spp. and *Kocuria* spp. were considered as contamination.

### Statistical Analysis and Comparisons

We summarized patient demographics using descriptive statistics: the mean (standard deviation) for continuous variables, and percentages for categorical variables. Statistical analyses was performed using SPSS Statistics for Windows, Version 28.0 (IBM Corp, Armonk, New York, USA).

#### Impact of Sonication Fluid Cultures When Using IDSA Criteria

We investigated the diagnostic value of sonication fluid by performing a head-to-head comparison of sonication fluid with intraoperative tissue culture (referred as tissue culture from now on) for each patients fulfilled 2012 IDSA criteria [11]. As the IDSA criteria include tissue culture but not sonication fluid cultures, a head-to-head comparison of the 2 methods enables the evaluation of their clinical value in a theoretical scenario where tissue cultures were substituted by sonication cultures. We calculated the number of cases in which sonication culture alone: (i) would not alter patient management (ie, antibiotic selection) as the results were fully concordant with tissue cultures, (ii) lead to undertreatment since sonication missed a virulent microorganism detected in tissue culture, (iii) could have potential value for patient's management due to detection of low-virulence micro-organism not identified in tissue culture, (iv) had clear additional value as it detected an additional virulent microorganism alongside another similar microorganism found tissue cultures, and (v) might cause ambiguity or unclear value for patient management due to the detection of a contaminant or a different micro-organism than that found in tissue culture.

The comparison between sonication fluid culture and tissue culture was stratified based on whether patients met the IDSA diagnostic criteria for PJI through clinical criteria alone or through microbiological findings. Clinical criteria include the presence of a sinus tract, or visible purulence around the prosthesis. In contrast, microbiological criteria are met by either (i) the isolation of at least one virulent microorganism from a tissue or synovial fluid culture, or (ii) the recovery of the same low-virulence microorganism from at least 2 specimens—either 2 separate tissue cultures, or 1 tissue and 1 synovial fluid culture. In cases meeting clinical criteria, cultures are not required to establish PJI diagnosis but are essential to select antibiotic therapy. By contrast, in patients meeting microbiological criteria, cultures are essential both to establish the diagnosis and to guide antimicrobial therapy.

#### Impact of Sonication Fluid Cultures When Using EBJIS Criteria

Unlike the IDSA criteria, the EBJIS definition of PJI includes sonication fluid culture alongside intraoperative tissue and synovial fluid culture [12]. The EBJIS criteria further stratified the PJI cases into confirmed and likely infections. Infection is confirmed if any of the following were present: sinus tract, leukocyte count >3.000/µl, polymorphonuclear leukocytes >80%, positive alfa-defensin in synovial fluid, ≥two positive samples (fluid and tissue) with the same microorganism, >50 CFU/mL of any organism in sonication fluid, or ≥ 5 neutrophils in ≥5 fields on histology analysis or presence of visible microorganisms. Infection is classified as likely when 2 of the EBJIS minor criteria were fulfilled. The cutoff of positive sonication fluid as minor criterion is set between 1 and 10 CFU/mL.

To evaluate the contribution of sonication fluid culture to the EBJIS definition, we stratified confirmed EBJIS cases based on whether they met the sonication criteria of >50 CFU/mL alone, or fulfilled other criteria. For cases that were based solely on meeting the >50 CFU/mL sonication criterion, we calculated the number of cases in which sonication identified microorganism that were not detected by tissue culture (ie, sonication fluid culture results had impact on antibiotic selection), or identified different microorganism than tissue culture (ie, sonication fluid culture caused ambiguity for antibiotic selection). In EBJIS confirmed criteria, missing organisms in sonication fluid culture was considered as irrelevant as the relevant microorganism has been detected via tissue culture. The same calculation was also performed for patients classified as having PJI based on EBJIS criteria other than sonication positive culture >50 CFU/mL.

Furthermore, for EBJIS likely cases, we calculated the number of cases based on the same microorganism in both sonication fluid culture (at the quantity of 1 to 50 CFU/mL) and tissue culture.

## RESULTS

### Demographic

A total of 429 patients were included, with a mean age (SD) of 68 (11) years, and 279 (65%) of them were male. Most patients (n = 381, 88.8%) had symptoms for longer than 4 weeks. Among the included patients, 246 (57.3%) underwent hip explantation, and 183 (42.7%) underwent knee explantation. Applying IDSA PJI criteria, 95 patients had definitive PJI, and 113 patients had confirmed PJI infection according to EBJIS criteria.

### Impact of Sonication Fluid Culture on IDSA Criteria

**Figure 1.**
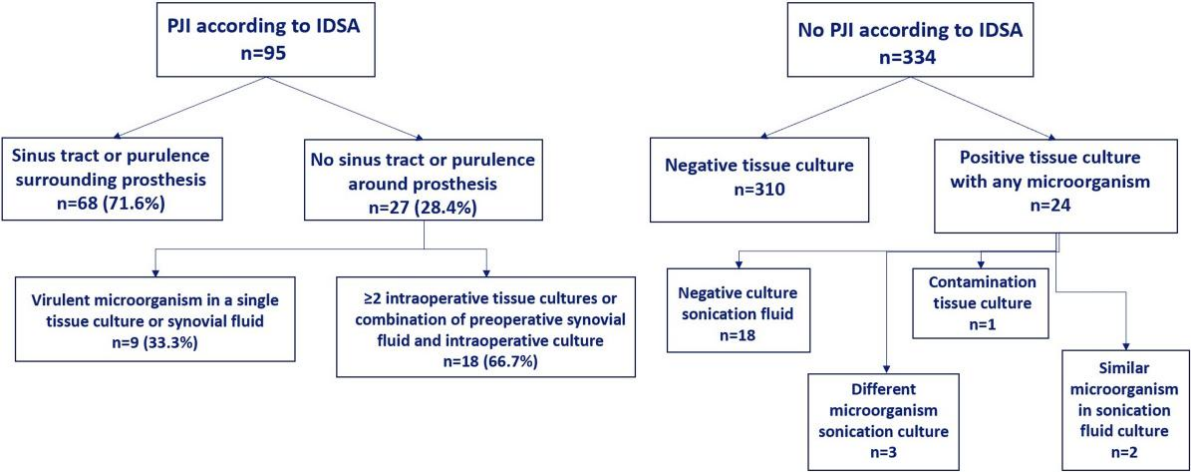
PJI cases according to IDSA criteria. Abbreviations: IDSA, Infectious Diseases Society of America; PJI, periprosthetic joint infection.

The breakdown of PJI cases according to IDSA criteria based on clinical criteria only (sinus tract or purulence surrounding the prosthesis or microbiology criteria) and no PJI are presented in Figure 1. Among the 95 PJI cases defined using IDSA criteria, sonication fluid culture would not alter patient management (ie, antibiotic selection) as the results were fully concordant with tissue cultures in 61 (64.2%) cases (sum of cases with superscript ‘a’ in Table 1). Sonication fluid had clear additional value as it detected an additional virulent microorganism alongside another similar microorganism found tissue cultures in 8 cases (8.4%, sum of cases with superscript ‘d’) and might have potential value for patient's management due to detection of low-virulence microorganism not identified in tissue culture in 12 (12.6%) cases (sum of cases with superscript ‘c’ ). In contrast, if sonication fluid cultures had been performed without other cultures, it would lead to undertreatment since it missed a virulent microorganism detected in tissue culture 11 (11.6%) cases (sum of cases with superscript ‘b’). It might cause ambiguity in patient management due to the detection of a contaminant or a different microorganism than that found in tissue culture in 3 of 95 (3.2%) PJI cases as determined by the IDSA criteria (sum of cases with superscript ‘e’).

**Table 1.** Comparison of Sonication Fluid Culture Results to Intraoperative Tissue Culture Among Patients With Prosthetic Joint Infection According to Infectious Diseases Society of America (N = 95)

	Acute	Chronic	Total (%)
Sinus tract or purulence around prosthesis (n = 68)			
Polymicrobial tissue culture (n = 14)			
Similar polymicrobial microorganisms as tissue cultures^a^	0	5	5 (7.3)
Miss a virulent microorganism while positive polymicrobial culture in tissues^b^	1	4	5 (7.3)
Add another virulent microorganism other than already cultured in tissues^d^	3	0	3 (4.4)
Add another virulent microorganism, but miss a low-virulence organism^d^	0	1	1 (1.5)
Monomicrobial tissue culture (n = 44)			
Similar virulent microorganism as in tissue cultures^a^	6	22	28 (41.2)
Similar low-virulence microorganism as in tissue cultures^a^	2	6	8 (11.8)
Miss a virulent microorganism^b^	0	3	3 (4.4)
Different virulent microorganism in monomicrobial tissue culture^e^	1	0	1 (1.5)
Different low-virulent microorganism in monomicrobial tissue culture^e^	0	1	1 (1.5)
Add a virulent microorganism to already cultured virulent microorganism in tissue culture^d^	0	1	1 (1.5)
Add a low-virulence microorganism to already cultured virulent microorganism in tissue culture^c^	0	2	2 (3.0)
Negative culture tissue (n = 10)			
Sonication also negative^a^	2	4	6 (8.8)
Add a virulent microorganism while tissue culture was negative^d^	1	1	2 (2.9)
Add a low-virulence microorganism while tissue culture was negative^c^	0	2	2 (2.9)
Positive tissue cultures or synovial fluid with virulent microorganism without sinus tract or purulence around prosthesis (n = 9)			
Similar virulent microorganism as in tissue cultures^a^	3	4	7 (63.6)
Similar virulent and low pathogen as in tissue cultures^a^	0	1	1 (9.1)
Add another virulent and a low-virulence microorganisms^d^	0	1	1 (9.1)
Miss two virulent microorganisms^b^	0	0	1 (9.1)
Miss a virulent microorganism^b^	0	0	1 (9.1)
≥ 2 positive tissue cultures or the combination between tissue culture and synovial fluid are positive with a low-virulence microorganism microorganisms (n = 18)			
Similar low-virulence microorganism^a^	1	8	6 (37.4)
Add other virulence microorganism^d^	1	0	1 (6.3)
Add other low-virulence microorganism^c^	0	7	8 (50.0)
Miss a virulent microorganism^b^	0	0	1 (6.3)
Contaminated sonication fluid^e^	1	0	1 (6.3)

^a^Performing sonication fluid culture only had no impact on antibiotic selection.

^b^Performing sonication fluid culture only may result in under treatment.

^c^Performing sonication fluid culture only could have potential value for patient's management due to detection of low-virulence microorganism.

^d^Performing sonication fluid culture only had clear additional value for management of the patient, and.

^e^Performing sonication fluid culture only may lead to clinical ambiguity.

In the group of patients without PJI based on IDSA criteria (n = 334), sonication cultures could potentially add 2 cases (0.6%) due to identification of similar low-virulence microorganisms as the tissue culture.

### Impact of Sonication Fluid Culture on EBJIS Criteria

**Figure 2.**
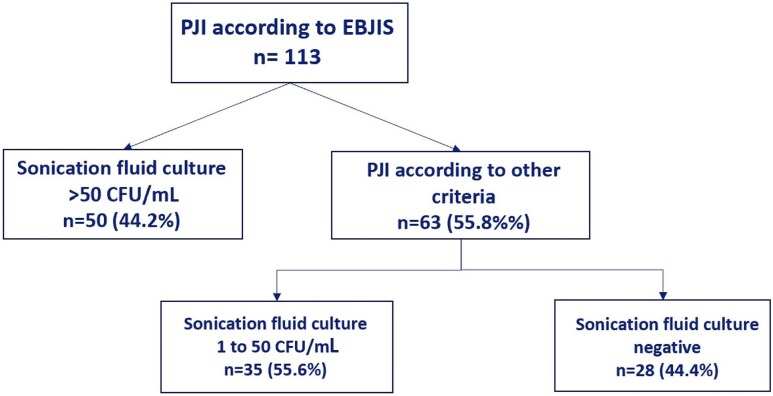
PJI cases according to EBJIS criteria. Abbreviations: EBJIS, European Bone and Joint Society; PJI, periprosthetic joint infection.

Using EBJIS criteria, 118 patients were deemed as likely infection and 113 patients as confirmed infection (Figure 2).

Among the confirmed cases, 50 out of 113 cases (44.2%) were based on meeting positive sonication fluid culture >50 CFU/mL criterion only. In 10 of those 50 patients (20%), sonication fluid culture had impact on antibiotic selection due to additional identification of another low-virulent microorganism, different than low-virulent microorganism detected in both tissue and sonication (Table 2). Among 63 patients who met EBJIS confirmed criteria based on other criteria than sonication >50 CFU/mL criterion, 7 patients (11.1%), sonication culture added microorganism next to microorganisms found in tissue culture (Supplementary Table).

**Table 2.** Comparison of Sonication Fluid Cultures With Tissue Cultures Among Patients Meeting the Sonication Fluid Criterion of >50 CFU/mL According to EBJIS Confirmed Criteria (N = 50)

	Acute (n = 8)	Chronic (n = 42)	N (%)
Exactly similar microorganisms	**6**	**28**	**34 (68%)^b^**
Similar polymicrobial virulent	0	1	1
Similar polymicrobial virulent and low-virulent	0	3	2
Similar monomicrobial low-virulent	1	3	4
Similar monomicrobial virulent	5	21	26
Missing monomicrobial virulent	**1**	**4**	**5 (10%)^b^**
Identification of another low-virulent microorganism, different than low-virulent microorganism detected in both tissue and sonication	**1**	**9**	**10 (20%)^a^**
Identification of different microorganism (n = 2, 4%)	**0**	**1**	**1 (2%)^c^**

Abbreviations: CFU, colony-forming units; EBJIS, European Bone and Joint Society.

^a^Sonication fluid culture results had impact on antibiotic selection.

^b^Sonication fluid culture results had no impact on antibiotic selection.

^c^Sonication fluid culture caused ambiguity for antibiotic selection.

None of the 11 patients with EBJIS likely criteria is based on similar positive sonication culture and a single positive tissue culture.

## DISCUSSION

In this study, we evaluated the impact of sonication fluid cultures on the diagnosis and management of PJI. We used a different approach from previous diagnostic accuracy studies, which primarily focused on calculating sensitivity and specificity of sonication fluid culture.

We used both the IDSA and EBJIS criteria, as they are widely used and because they differ in how they incorporate sonication fluid culture results, allowing for a distinct comparative analysis.

The IDSA criteria include microbiological criteria of positive tissue cultures but do not incorporate sonication fluid culture. By comparing sonication culture and tissue culture results for each patient, we could assess whether sonication identified the same microorganism, detected additional ones, or missed a microorganism found in tissue. This approach allowed us to estimate the potential change in patient management (ie diagnosis and change in antibiotic selection) in a theoretical scenario if only sonication fluid culture was available. We found that in approximately two-thirds of PJI cases, relying solely on sonication fluid culture would not have altered the diagnosis or antibiotic selection. However, in 8% of the cases, sonication detected an additional virulent microorganism that could influence antibiotic selection.

Unlike the IDSA criteria, the EBJIS criteria include sonication fluid culture alongside positive tissue and synovial fluid cultures as part of the diagnostic definition. We found that nearly half of the confirmed PJI cases were based on the criterion of positive sonication fluid culture with >50 CFU/mL. Because EBJIS includes both sonication and tissue microbiology criteria, a microorganism missed by sonication does not pose a risk of undertreatment if it is already detected in tissue. In contrast, any additional microorganism detected by sonication fluid culture may change patient management. We found that patient management could change in 20% of cases due to the identification of an additional microorganism that was not detected in tissue cultures. This a relatively high proportion. Comparison of this observation with previous studies is challenging, as most were diagnostic accuracy studies that evaluated sonication in terms of test performance—focussing on sensitivity and specificity—without examining its impact on diagnosis and management of the patient [15].

As with the IDSA and EBJIS criteria, sonication fluid culture rarely contributed to identifying new PJI cases when only a single positive clinical, radiological, or microbiological criterion was present and additional criterion was required. This finding contrasts with another study of 289 patients, which reported that 8 additional cases were diagnosed due to sonication fluid culture results [16].

The information from this study can help clinicians estimate the potential change in patient management when sonication culture is used. Our results also provide a basis for evaluating whether sonication is needed in a clinical microbiology laboratories, given that it is both laborious and is costly. In our European survey, we found that half of the laboratories did not offer sonication fluid cultures [9].

This study has several strengths. We were able to specifically assess the impact of quantifying colony growth in sonication fluid cultures using both the IDSA and EBJIS criteria. Notably, 77% of patients had at least 3 tissue cultures, as recommended by the IDSA guideline. The EBJIS criteria explicitly defines colony counts thresholds: growth of >50 CFU/mL of any microorganism is considered a criterion for confirmed PJI, whereas growth of 1 to 50 CFU/ mL requires an additional positive criterion to suggest likely infection. Such detailed consideration is often lacking in other studies. We also evaluated polymicrobial infections by directly comparing sonication fluid and tissue cultures on a patient-by-patient basis. This approach addresses a common challenge in calculating sensitivity or specificity of sonication fluid cultures in cases involving polymicrobial growth, which can complicate interpretation in many studies [17]. However, our study has some limitations. The study period spans several years, during which clinical or laboratory procedure may have changed. Nevertheless, interim analysis showed no significant variation in the number of tissue samples submitted, and culture protocols remained consistent throughout the study period. Another limitation is that we did not assess whether identifying additional microorganisms through sonication—those not detected in tissue cultures—actually changed antibiotic selection. It is possible that the additional organisms were susceptible to the same antibiotics used for those identified in tissue cultures. Conducting such an analysis would be complex and labor-intensive. Finally, we included only explanted implants and excluded mobile components removed during debridement, antibiotics, and implant retention procedures. These were excluded because sonication of mobile parts is difficult to standardize due to variability in size and material.

In conclusion, our study shows that performing sonication fluid culture might change patient management between 8% and 20% of PJI cases.

## Supplementary Material

ciaf391_Supplementary_Data

## Supplementary Data

Supplementary materials are available at *Clinical Infectious Diseases*  online. Consisting of data provided by the authors to benefit the reader, the posted materials are not copyedited and are the sole responsibility of the authors, so questions or comments should be addressed to the corresponding author.

## Note

***Financial support***. Next to routine salaries, no additional funding was received in performing this study.
