# Supplementary material for: Impact of Sonication Fluid Cultures on Prosthetic Joint Infection Diagnosis and Management: A Microbiology-Driven Evaluation Using Infectious Diseases Society of America and European Bone and Joint Infection Society Criteria
Source: Clin Infect Dis. 2025 Jul 18;81(5):e263–8. doi: 10.1093/cid/ciaf391 (PMC12728276; doi:10.1093/cid/ciaf391)
Supplement: ciaf391_Supplementary_Data [file ciaf391_supplementary_data.docx]

**Supplementary Table.** Comparison of sonication fluid cultures with tissue cultures among patients meeting other criteria than sonication fluid criterion of >50 CFU/ml according to EBJIS confirmed criteria (n=68)

| **Sonication culture positive, 1 to 50 CFU/ml** | **Acute (n=13)** | **Chronic (n=22)** | **35 (30.9%)** |
| --- | --- | --- | --- |
| Exactly similar microorganisms | **8** | **17** | **25** |
| Similar polymicrobial virulent | 0 | 0 | 0 |
| Similar polymicrobial virulent and low-virulent | 0 | 0 | 0 |
| Similar monomicrobial low-virulent | 4 | 9 | 13 |
| Similar monomicrobial virulent | 4 | 7 | 11 |
| Missing monomicrobial virulent | **0** | **1** | **1** |
| Identification of another micro-organisms | **4** | **3** | **7** |
| Adding low virulent next to polymicrobial pathogen | 1 | 0 | 1 |
| Add low virulent | 2 | 2 | 4 |
| Add virulent | 1 | 1 | 2 |
| Identification of different micro-organism or contamination | **1** | **2** | **3** |
| Contaminant in sonication fluid culture | 1 | 0 | 1 |
| Add virulent, but missing low-virulent | 0 | 1 | 1 |
| Different low virulent | 0 | 1 | 1 |
| **Sonication culture negative** | **5** | **23** | **28 (24.9)** |
